# The Gut–Ear Axis: From Dysbiosis to Auditory and Vestibular Disorders

**DOI:** 10.3390/ijms27177523

**Published:** 2026-08-22

**Authors:** Yutian Li, Xinyu Shi, Xiaozhou Liu, Yu Sun

**Affiliations:** 1 Department of Otorhinolaryngology, Union Hospital, Tongji Medical College, Huazhong University of Science and Technology, Wuhan 430022, China; 2 Institute of Otorhinolaryngology, Union Hospital, Tongji Medical College, Huazhong University of Science and Technology, Wuhan 430022, China; 3 Hubei Province Clinic Research Center for Deafness and Vertigo, Wuhan 430022, China; 4 Hubei Province Key Laboratory of Oral and Maxillofacial Development and Regeneration, Wuhan 430022, China

**Keywords:** gut microbiota, gut dysbiosis, gut-ear axis, audiovestibular disorders

## Abstract

With further research on the relationship between gut microbiota and human health, discussions on various gut-X axis have been increasingly prevalent. Evidence indicates that microbiota dysbiosis is closely linked to the onset and progression of audiovestibular disorders and the gut-ear axis has gradually been recognized as a vital systemic regulatory pathway. This article systematically reviewed the interaction mechanisms between microbiota dysbiosis and audiovestibular diseases, intervention strategies, research limitations and future perspectives. This axis functions mainly through immune-mediated barrier damage, metabolic disorder and neurotransmitter crosstalk. Modulation of the gut microbiota can alleviate symptoms of certain audiovestibular disorders. This review aims to provide novel insights for the pathogenesis, intervention and clinical management of audiovestibular disorders.

## 1. Introduction

The gut microbiota is a complex and dynamic microbial community that colonizes the mammalian gastrointestinal tract [1,2]. Mounting clinical and preclinical evidence demonstrates that gut microbiota actively engages in bidirectional crosstalk with the central nervous system and multiple extraintestinal organs, rather than functioning independently in the intestinal lumen [3,4,5]. Gut microbial dysbiosis triggers persistent systemic low-grade inflammation and oxidative stress, accompanied by the release of numerous microbial metabolites and pro-inflammatory mediators into peripheral circulation [6,7]. These gut-derived circulating factors can target distant organs and even penetrate the blood–brain barrier (BBB), disrupting central nervous system homeostasis and contributing to the development of various neurological and systemic disorders [8,9].

Similar to the central nervous system, the inner ear is traditionally regarded as a sterile organ under healthy conditions protected by a specialized vascular barrier, the blood-labyrinth barrier (BLB) [10,11,12]. The BLB shares high structural and functional homology with the BBB; both barriers insulate local microenvironments from systemic blood circulation, limit aberrant substance exchange, and sustain regional ionic and immune homeostasis [13,14]. Although the inner ear has traditionally been regarded as a sterile environment under healthy conditions, recent 16S rRNA sequencing-based studies have revealed that the external auditory canal and middle ear harbor distinct resident microbial communities. The healthy external auditory canal is predominantly colonized by *Staphylococcus* (particularly *S. auricularis* and *S. capitis*), *Cutibacterium*, and *Corynebacterium* species, along with other skin-associated bacteria [15,16]. These findings indicate that the ear is not entirely sterile and harbors resident microbial communities that may contribute to local immune modulation. However, the presence of these local communities does not diminish the importance of systemic signals from the gut; the BLB renders the inner ear vulnerable to circulating microbial metabolites and inflammatory mediators, providing a biological foundation for the gut-ear axis, a novel regulatory pathway that affects inner ear physiology and pathology [17,18,19].

**Figure 1:**
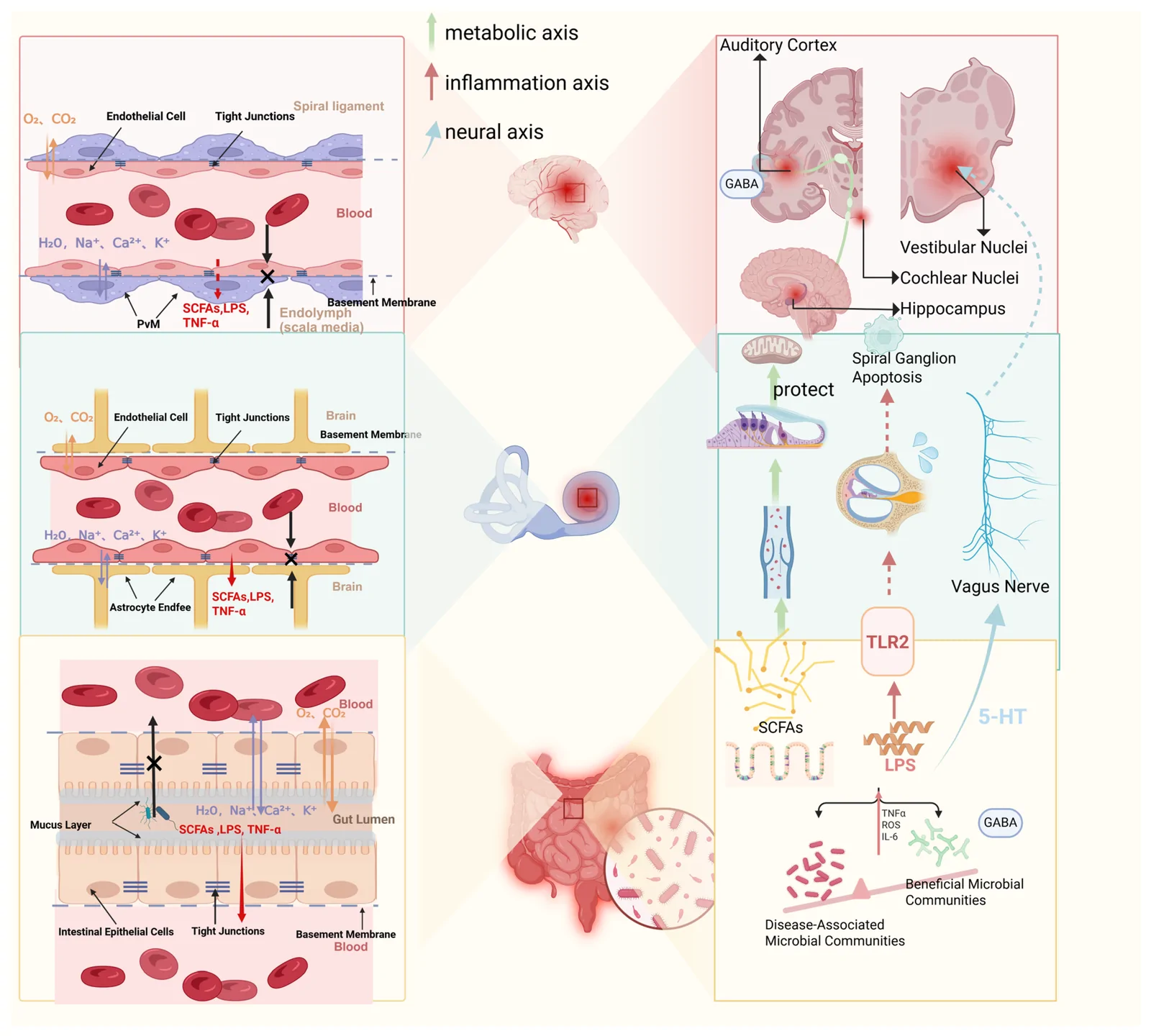
Schematic illustration of the gut–ear axis: from intestinal barrier to auditory and vestibular systems. This schematic integrates the intestinal barrier, the BBB and the BLB on the left panel. The three proposed signaling routes, including metabolic (green), inflammatory (red), and neural (blue), are shown on the right panel. Metabolic axis (Green arrows): Microbial Short Chain Fatty Acids (SCFAs) enter systemic circulation, enter the cochlea through the bloodstream to produce ROS and affect the function of hair cells; Inflammation axis (Red arrows): Lipopolysaccharide (LPS) from Disease-Associated Microbial Communities activates Toll-like Receptor 2 (TLR2), triggering pro-inflammatory cascades that disrupt the blood-labyrinth barrier and promote neuronal damage; Neural axis (Blue arrows): The vagus nerve relays gut-derived signals to cochlear nuclei and vestibular nuclei, modulating central auditory processing and balance coordination; Solid arrows: indicate pathways with direct experimental evidence in the auditory system; Dashed arrows: denote hypothesized routes that are well-established in the gut–brain axis but await direct validation in the inner ear. Abbreviations: TLR2, Toll-like Receptor 2; SCFAs, Short-Chain Fatty Acids; LPS, Lipopolysaccharide; TNF-α, Tumor Necrosis Factor-alpha; PvM, perivascular macrophage-like melanocyte; 5-HT, serotonin; GABA, γ-aminobutyric acid.

Audiovestibular diseases, including hearing loss (HL), tinnitus, Ménière’s disease (MD) and benign paroxysmal positional vertigo (BPPV), represent a large group of widespread sensory diseases [20,21,22,23,24]. Their complex pathogenesis and heterogeneous clinical manifestations pose major challenges to current diagnosis and treatment, making relevant mechanistic research an urgent clinical need [20,21,22,23,24]. Traditional research has predominantly focused on local pathological and molecular alterations within the auditory and vestibular systems, including the cochlea, vestibulocochlear nerve, brainstem nuclei, and cerebral cortex [25,26]. However, such local-centric perspectives overlook systemic regulatory mechanisms, failing to fully explain the clinical heterogeneity and poor treatment responses observed in refractory audiovestibular diseases [26,27]. Recent studies have preliminarily correlated gut dysbiosis-induced inflammation, metabolic dysfunction, and neurotransmitter disturbance with the progression of audiovestibular disorders, with probiotic intervention showing tentative therapeutic potential [17,18,19] (Table 1, Table 2 and Figure 1). Although the gut-ear axis has been conceptually proposed, the precise mechanisms underlying gut dysbiosis-mediated audiovestibular pathogenesis remain poorly defined, and relevant evidence for microbiome-targeted therapy remains fragmented and insufficient [3,4,5].

This review systematically summarizes the involvement of gut dysbiosis in the pathogenesis of audiovestibular diseases (Table 2). Meanwhile, this review proposes the notion of potential microbiome-related biomarkers and prospective precision-targeted intervention strategies, providing novel insights for the prevention and clinical management of audiovestibular diseases.

## 2. Hearing Loss

Hearing loss affects 20% of the global population [59]. Its common causes are currently recognized to include genetics, aging, noise exposure, viral infection, and the use of ototoxic drugs [60,61,62,63,64]. Existing treatment dilemmas include but are not limited to the radical cure of sensorineural hearing loss [65,66,67], the limited function and the high cost of cochlear implantation lead to low utilization [68,69,70,71], and breakthroughs have been made in gene therapy, but the safety and efficiency of drug delivery remain major concerns [72,73]. Given that the current pharmacy has difficulty crossing the blood-labyrinth barrier and entering the inner ear [74,75,76,77,78], the gut microbiota, which can influence the body by altering the barrier, may represent a potential target for hearing loss research, though its clinical relevance remains to be established.

Animal models have provided mechanistic insights. Pisani et al. (2025) [19] transplanted fecal microbiota from active (aUC) or remission ulcerative colitis (rUC) patients into DSS (dextran sulfate sodium)-treated mice. aUC FMT (active Ulcerative Colitis Fecal Microbiota Transplantation) elevated ABR thresholds by 10–15 dB with cochlear inflammation and BLB disruption, whereas rUC FMT (remission Ulcerative Colitis Fecal Microbiota Transplantation) showed no significant effect and appeared relatively protective. Meanwhile, this study also found altered levels of MyD88 (Myeloid Differentiation Primary Response 88) in the cochlea of mice with gut microbiota dysbiosis, which is one of the most important adaptor proteins in TLR signaling, activating downstream molecules such as NF-κB (Nuclear Factor Kappa-Light-Chain-Enhancer of Activated B Cells) [79]. Additionally, in the cochlea of this group, proteins important for the BLB structure, such as ZO-1 and occludin, were significantly reduced. This was accompanied by signs of vascular extravasation, changes in pericyte structure, and infiltration of CD45-labeled leukocytes and IBA-1-labeled macrophages. These findings suggest that gut dysbiosis may contribute to cochlear dysfunction in animal models, even in the absence of direct acoustic or ototoxic insults, with manifestations including disruption of the blood-labyrinth barrier, increased oxidative stress, and local inflammation. The association between noise exposure and gut microbiota has been investigated in rodent models with consistent results. Li et al. exposed rats to chronic noise at 95 dB or 105 dB SPL for 30 days and found dose-dependent gut microbial alterations: 6 genera, including *Romboutsia* and *Turicibacter,* were significantly changed in the lower-noise group, whereas 14 genera were altered in the higher-noise group, including *Akkermansia* and *Corynebacterium_1,* accompanied by significant differences in both α- and β-diversity [80]. Sato et al. independently reproduced this observation in C57BL/6J mice exposed to 95 dB SPL for 30 days, which showed distinct β-diversity clustering and genus-level shifts including *Lactobacillus*, *Blautia*, *Oscillospiraceae*, *Colidextribacter*, and *Clostridia UCG-014* [81]. These convergent findings from two independent laboratories indicate that chronic noise exposure consistently alters gut microbial community structure, though the functional implications of these shifts for cochlear health remain to be determined.

Human genetic and observational studies have extended these findings. MR studies have identified causal associations between certain gut microbial genera and SNHL [28,41]. Some genera have been associated with protective effects, including *Lachnospiraceae UCG001* and *Intestinimonas*, while others may increase the risk, including *Rikenellaceae RC9* gut group, *Eubacterium hallii* group, and *Porphyromonas*. Almazán-Catalán et al. conducted a study involving a young population; individuals with hearing loss showed a higher relative abundance of *Faecalibacterium prausnitzii* in their fecal microbiota, and this was associated with certain antibiotic resistance genes, hinting at a potential link between the genome of intestinal drug resistance and hearing [82].

Furthermore, although Kociszewska et al. [83] have proposed the hypothesis that the gut–ear axis may influence age-related auditory decline in mice through inflammatory, immune, and metabolic pathways, the relationship between gut microbiota and presbycusis has not been adequately validated in human populations. Yang et al. found that germ-free mice receiving fecal microbiota transplantation exhibited auditory aging effects related to microbiota, suggesting that age-related hearing loss may be influenced by gut microbes [84]. Li et al. integrated mouse cochlear omics data with human MR analysis using the GEO database and proposed a hypothetical model of the “*Roseburia*–FPR2 (Formyl Peptide Receptor 2)–taurochenodeoxycholic acid” regulatory network, providing a novel framework for predicting the potential role of the gut–ear axis in age-related hearing loss [16].

Dietary and metabolomic studies further suggest modifiable pathways. The gut microbiota can also be influenced and changed by different dietary patterns, thereby regulating the cochlea. Chan et al. found that high-fat diets induce inflammatory changes in the cochlea, upregulating proteins such as ICAM1, IL-6Rα, and TLR2 and recruiting macrophages [45,85,86,87]. This suggests a transient inflammatory phase aimed at mitigating long-term damage [88,89]. Maintaining gut health through dietary approaches, such as omega-3 supplementation or Mediterranean diets, has been associated with lower hearing loss risk in observational studies, suggesting a hypothesis that warrants testing in interventional trials [39,46,47,48,49,50]. Meanwhile, MR studies have identified specific food preferences and gut bacterial species that significantly influence SNHL risk, reinforcing the potential protective role of diet and the microbiome [28,41,90]. At the same time, SCFA-producing anaerobic bacteria have been associated with lower SNHL risk in MR studies. Whether targeted enhancement of these microbes confers protection in humans remains to be determined [91].

In summary, animal experiments demonstrate that gut dysbiosis, whether induced by high-fat diet or fecal microbiota transplantation, can promote cochlear inflammation and elevate auditory thresholds. MR analyses have identified several candidate genera associated with SNHL risk, suggesting potential causal relationships. However, direct evidence in humans is scarce, and the functional pathways from gut-derived signals to cochlear damage have not been directly traced. The link between gut microbiota and hearing loss is supported by convergent evidence from animal models and MR studies, yet significant gaps remain. Whether microbiome-targeted interventions confer measurable hearing benefits in clinical populations remains an open question that requires rigorous testing.

## 3. Otitis Media

Otitis media (OM) is understood as an inflammatory or infectious disease of the middle ear, mainly involving the middle ear cavity and its mucosal system [92]. Acute otitis media (AOM) affects 10.85% of the global population per year, with 51% of cases occurring in children, and 22.6% of these cases occur in children under five years of age [93]. However, misdiagnosis and overdiagnosis are very common, leading to unnecessary antibiotic use and increased drug resistance [64,94,95]. Biofilms and drug resistance make chronic cases difficult to cure, and some patients progress to severe complications [96,97]. However, high-quality clinical trials and unified guidelines are still insufficient [98,99]. As a disease caused by dysbiosis of the microecology in the middle ear and upper respiratory tract [100,101], the concept of the gut-ear axis introduces OM into an overall micro-ecosystem rather than a single pathogen, which brings new theories and targets.

Among the available evidence, the strongest support comes from randomized controlled trials and their meta-analyses. The role of probiotics in OM has been examined. A systematic review and meta-analysis including 16 RCTs with 4034 participants evaluated the effects of probiotics, prebiotics, or synbiotics on AOM [42,51]. No significant effects were observed on time to first episode, recurrence rate, or the need for antibiotic use. However, the overall risk of AOM was reduced by approximately 20% (RR 0.80, 95% CI 0.66–0.96), and the effect was influenced by intervention duration, age, and the number of strains used. In an RCT of antibiotic treatment for AOM, amoxicillin significantly increased the risk of gut colonization by resistant bacteria. Children who lacked *Blautia*, *Ruminococcus*, *Faecalibacterium*, *Roseburia*, or *Faecalitalea* were more susceptible to colonization by antimicrobial-resistant (AMR) bacteria, suggesting that certain key commensal gut bacteria are important for reducing post-antibiotic sequelae of AOM, including antibiotic-associated diarrhea (AAD), allergic reactions [102]. Oral probiotics combined with reduced-dose antibiotics may be more effective than full-dose antibiotics alone in AOM, with proposed potential mechanisms including inflammation reduction, SCFA production, and microcirculation maintenance.

Cross-sectional studies have explored gut–OM associations, though with limited findings. Population-based studies have shown a partial connection between the gut microbiota and OM. In a cross-sectional study of children with recurrent acute otitis media (rAOM) [103], the overall gut microbiota composition showed only limited differences between the rAOM group and healthy controls, with only six bacterial taxa, including *Veillonella*, *Lachnospiraceae*, *Bacteroides*, *Ruminococcaceae*, and *Blautia*, exhibiting differences. However, certain bacterial taxa were correlated with the number of episodes, among which *Turicibacter* showed the most prominent association with consecutive infection episodes, suggesting that specific bacteria may be linked to the frequency of disease recurrence.

Although the ear microbiome is less studied than the gut microbiome, it forms a stable and niche-specific microbial community after progressive colonization. Audiovestibular diseases are also closely associated with the ear microbiome. Under healthy conditions, the microbiome of the external auditory canal (EAC) is predominantly composed of skin commensal bacteria. Studies have demonstrated that the core genera consist of obligate or facultative anaerobes, including *Staphylococcus auricularis*, *Propionibacterium acnes*, *Alloiococcus otitis* and *Turicella otitidi* [104]. These microorganisms generally form stable biofilms and maintain local immune homeostasis. A 16S rRNA gene sequencing analysis of 50 healthy adults revealed that the EAC microbiome is dominated by *Corynebacterium* and *Staphylococcus*, while *Cutibacterium* presents high abundance in some individuals [105]. Traditionally, the healthy middle ear has been considered a sterile niche [106]. Nevertheless, recent findings have challenged this view. Through sterile sampling and metagenomic analysis of the middle ear cavity from patients undergoing cochlear implantation, researchers have detected reproducible microbial signals with low biomass, mainly comprising *oropharyngeal* and nasopharyngeal taxa such as *Streptococcus*, *Staphylococcus* and *Ralstonia* [107]. Notably, Chan et al. systematically compared the microbiomes of EAC lavage fluid, adenoid tissues and middle ear effusion, and confirmed that both the external auditory canal and adenoids serve as important microbial sources for middle ear effusion [108]. As for the inner ear, there is currently no direct evidence supporting stable microbial colonization within this compartment. Enclosed by rigid bony structures and protected by the BLB, the inner ear is theoretically maintained in a sterile state.

The classic bacterial triad of otitis media (OM) includes *Streptococcus pneumoniae*, *Moraxella catarrhalis* and non-typeable * Haemophilus influenzae* (NTHi) [29]. Studies on children aged 6–12 years have demonstrated a significant correlation between adenoid microbiota and chronic otitis media with effusion (COME), where *Streptococcus pneumoniae* and *Haemophilus influenzae* were prominent indicators. Alterations in the nasopharyngeal microbiome may modulate immune responses via spermidine and acetate production, accelerating COME progression [109]. NGS (Next-Generation Sequencing) reveals under-recognized taxa like *Alloiococcus otitidis* and *Turicella otitidis* in COME [110]. A stable upper respiratory microbiome dominated by *Corynebacterium* and *Dolosigranulum* correlates with lower OM risk, whereas *Haemophilus*/*Streptococcus* enrichment predicts instability [110]. This suggests that indigenous commensal bacteria, not limited to the gut microbiota, can regulate the progression of audiovestibular diseases. Moreover, it is worth noting that *Salivary streptococci*, a beneficial species colonizing the healthy nasopharynx, can strongly suppress the proliferation of multiple common pathogens related to otitis media [111,112]. In children with recurrent otitis media, there is no significant difference compared with healthy individuals, but some bacteria character, like *Turicibacter*, are connected to the frequency and severity of inflammation [56].

The evidence for gut microbiota involvement in otitis media is more established than for other audiovestibular disorders, largely due to multiple randomized controlled trials and meta-analyses. Probiotic supplementation appears to reduce AOM risk by approximately 20%, though effect sizes vary across studies and no consensus exists on optimal strains or dosing. Cross-sectional studies have yielded limited and inconsistent gut-OM associations, whereas the upper respiratory tract microbiome shows stronger and more direct links to OM pathogenesis. The contribution of gut microbiota to OM susceptibility may be indirect, possibly mediated through systemic immune modulation or antimicrobial resistance gene transfer, but this remains speculative and requires further mechanistic investigation.

## 4. Tinnitus

Tinnitus, the perception of a persistent ringing sound without an external source, affects approximately 10% to 30% of adults globally [113,114,115,116]. The causes of tinnitus are various and difficult to clarify clinically [117,118,119,120]. Therefore, the treatment of tinnitus mainly focuses on symptom control rather than etiological treatment [121,122,123]. The existing theories of the mechanism of tinnitus include abnormal discharge of peripheral nerves [124], plasticity changes in central nerves [125,126,127], and inflammation of the limbic system [128] and the central nervous system [129]. However, these theories cannot fully explain the mechanisms of tinnitus, such as tinnitus without hearing loss and the high clinical heterogeneity of tinnitus [33,58,114,118]. The gut microbiota represents a novel perspective that may help to expand the current conceptual framework of tinnitus and could provide new mechanistic candidates worthy of future exploration.

Cross-sectional multi-omics studies have screened candidate microbial and metabolic signatures. A study involving 70 patients with chronic tinnitus and 30 healthy controls found that patients with tinnitus had significant gut microbiota imbalance, including reduced overall diversity and increased ratio of *Firmicutes/Bacteroidetes* [32]. There was a decrease in a variety of beneficial bacteria, such as *Lactobacillus*, *Lactococcus*, *Akkermansia*, and some *Prevotella*, whereas there was an increase in opportunistic pathogens, such as *Aeromonas*, *Acinetobacter*, and *Rhodococcus*. In addition, serum metabolomics of chronic tinnitus patients showed that patients with tinnitus have extensive metabolic disorders, and the related pathways involve neuroinflammation, neurotransmitter activity and synaptic function. This study utilized a discriminative model established by differential microbiota and metabolites, and the diagnostic accuracy for tinnitus could reach 0.94–0.96, suggesting that these indicators may hold potential biomarker value pending prospective validation in larger cohorts. These findings from a cross-sectional study suggest that alterations in the gut microbiota may be associated with functional changes in brain and auditory pathways, potentially mediated through serum metabolites. However, whether these associations translate into a causal role in tinnitus pathogenesis requires prospective validation.

An MR study analyzed the relationship between 412 gut microbiota characteristics and the risk of tinnitus using GWAS (Genome-Wide Association Study) data [53]: an increased abundance of a certain genus of bacteria was associated with a reduced risk of tinnitus (OR 0.84).

Based on data from over 900,000 individuals in the UK Biobank, Fang et al. explored the mediating relationships among gut microbiota, brain functional connectivity, and tinnitus [54]. Resting-state functional MRI (rfMRI) revealed that patients with tinnitus exhibited reduced connectivity in the salience network, default mode network, and central executive network. Moreover, *Actinobacteria* and the *chorismate* biosynthesis I pathway were found to be associated with both tinnitus and brain connectivity; the increased abundance of actinomycetes phyla and classes is associated with an increased risk of tinnitus. These findings raised the possibility that the gut microbiota could influence tinnitus risk through alterations in brain network connectivity. This hypothesis warrants testing in longitudinal and intervention studies before therapeutic implications can be drawn.

Current evidence linking gut microbiota to tinnitus is predominantly correlational and derived from cross-sectional studies. Patients with tinnitus consistently show reduced gut microbial diversity and altered taxonomic composition, but whether these changes precede or follow tinnitus onset cannot be determined from existing data. MR analyses suggest potential causal signals for certain taxa, and neuroimaging studies have raised the possibility of brain-mediated pathways. However, the clinical utility of microbiota-based biomarkers remains uncertain, and no intervention study has convincingly demonstrated that modifying the gut microbiota alleviates tinnitus in humans. Prospective longitudinal studies are urgently needed to establish temporal directionality.

## 5. Vestibular Diseases

The vestibular diseases are classified into central and peripheral types [34]. This section mainly focuses on peripheral vestibular disorders, which mainly contain MD and BPPV. MD, characterized by episodic vertigo, hearing loss, and aural fullness, whose pathogenesis is closely related to membranous labyrinth hydrops, has limited treatment evidence [130,131,132]. BPPV is recognized as one of the most prevalent vestibular disorders [133] and is mainly believed to be a disease where otoliths fall off and enter the semicircular canals, causing abnormal mechanical stimulation [134]. The complex membranous labyrinth structure of the vestibule poses obstacles to research [135,136], while the indirect regulation of the gut-ear axis offers a theoretical advantage for reaching the vestibule [19].

Fumihiro et al. [34] analyzed intestinal flora samples of 10 MD patients and 11 healthy donors (HD), indicating significant negative correlations between MD patients’ disease duration and alpha diversity indexes. Moreover, the genus *Butyricicoccus* produces butyrate, one of the SCFAs [137], whose abundance is positively correlated with disease duration in MD patients [34]. Given that SCFAs are known to participate in the gut–brain axis, and it is hypothesized that they could play a role in the progression of MD, although direct evidence in vestibular disorders is lacking [138,139].

To investigate whether gut microbiota contributes to the comorbidity of anxiety and vestibular dysfunction, Li et al. established an UL mouse model to simulate peripheral vestibular dysfunction [55]. Within 7 days post-surgery, 16S rRNA sequencing and liquid chromatography–mass spectrometry (LC-MS) revealed two notable findings. First, consistent with Fumihiro et al.’s observations in Ménière’s disease patients, UL mice showed a >40% reduction in SCFA-producing genera, including *Lachnospiraceae NK4A136*, *Roseburia*, and *Odoribacter*. Second, in contrast to the negative correlation between disease duration and α-diversity reported by Fumihiro et al., UL mice unexpectedly exhibited higher gut microbial community diversity than sham-operated controls. Additionally, the anxiety-associated genus *Parasutterella* increased 3-fold in UL mice [140].

A bidirectional MR conducted by Rong et al. (2024) investigates the causal relationships between gut microbiota and BPPV [56]. Among ten taxa, there were two taxa with odds ratios (OR) greater than one. In contrast, eight taxa had an OR of less than one, including four families, three genera, and one order. Mediation analysis of BPPV revealed that major depression, obesity, and HbA1c were key mediators consisting of the *class Lentisphaeria*, the *order Victivallales*, the *genus Bifidobacterium*, the *family Bifidobacteriaceae*, and the *order Bifidobacteriales* between specific taxa and BPV.

Several studies have been conducted on Vestibular migraine (VM). In a VM-like rat model established using nitroglycerin and rotational stimulation [58], 16S sequencing revealed a decreased evenness of the gut microbiota; *Lactobacillus* and *Bifidobacterium* were increased, whereas *Lachnospiraceae NK4A136 group* and others were markedly decreased. Meanwhile, the serum metabolome of VM-like rats showed alterations in amino acid metabolism, sphingolipid signaling, and vitamin pathways. The authors suggest that this ‘gut dysbiosis and metabolic disturbance’ participates in the pathogenesis of VM via the gut–brain axis and may serve as a potential intervention target. However, Webster et al. explored the effects of probiotics on the comprehensive vertigo score at two-month and four-month follow-ups, and the evidence is very uncertain [57]. At two-month follow-up, probiotics may have little or no effect on the number of vertigo episodes. The difference at four months is also negligible, but the evidence is very uncertain. Finally, when assessed at either the two-month or four-month follow-up, probiotics may have little or no effect on disease-specific health-related quality of life, although the evidence from this study is of low certainty.

Abnormal audiovestibular function may also affect microbial homeostasis, commonly characterized by reduced microbial structural diversity and aberrant abundance of specific genera [58]. After intratympanic administration of antibiotics for otitis media, opportunistic pathogens in the gut microbiota also proliferated with increased relative abundance. This further reveals the subtle bidirectional communications between the gut and ear.

The evidence base for gut microbiota involvement in vestibular disorders remains the weakest among the diseases reviewed. Available data are limited to small cross-sectional studies and animal models, with sample sizes typically under 30 participants and no replication cohorts. While some correlational patterns have been reported, such as the association between *Butyricicoccus* and MD duration, these findings require validation in larger, well-characterized populations. The causal relationships inferred from MR studies are informative but do not replace experimental evidence. Whether gut-derived metabolites or inflammatory signals reach the vestibular system in sufficient concentrations to affect balance function is entirely unknown and represents a major knowledge gap.

## 6. Limitations and Further Prospects

The gut microbiota participates in the pathogenesis of audiovestibular disorders mainly through four interrelated pathways. First, an imbalance in microbial metabolites plays a pivotal regulatory role. Microbial metabolites, particularly SCFAs, play a protective role by preserving cochlear mitochondrial homeostasis and reducing oxidative stress [34]. In contrast, pro-inflammatory metabolites such as LPS would trigger Toll-like receptor-mediated inflammatory injury in the cochlea [58]. Second, inflammation derived from the gut compromises the blood-labyrinth barrier, exacerbating cochlear inflammation and neuronal damage [18,83]. Third, gut microbiota modulates the synthesis and secretion of neurotransmitters, including GABA and serotonin, which affect auditory signaling transduction and central vestibular compensation, closely linked to tinnitus and balance dysfunction [53,59,141]. Fourth, shared genetic pathways link the development of the enteric nervous system and inner ear morphogenesis, further solidifying the gut-ear connection [16].

Notably, several limitations inherent in current investigations should be duly recognized. Primarily, the majority of available evidence originated from cross-sectional surveys and preclinical animal experiments. Given the substantial interspecific divergence in microbial composition, a threefold increase of *Parasutterella* observed in murine labyrinthectomy has not yet been confirmed in humans [55]. Clinically, human trials were generally constrained by limited sample size and heterogeneous enrollment standards, which impede quantitative comparison and result consolidation [34]. Methodologically, conventional 16S rRNA sequencing cannot achieve strain-level discrimination, nor can it characterize disease-relevant viral and fungal communities, which restricts the mechanistic exploration [110]. Furthermore, most underlying regulatory pathways are deduced from indirect evidence. For instance, although intestinal Bifidobacterium is capable of synthesizing GABA, definitive evidence verifying the translocation of gut-derived GABA into the central auditory pathway and its direct modulatory role in tinnitus pathogenesis remains lacking [29,91]. Direct causal validation in humans is almost unachieved. This problem stems from the complex transmission of the gut-ear axis, covering the intestinal lumen, epithelial barrier, circulatory metabolism, and blood-labyrinth barrier penetration. In vivo tracking of specific microbial metabolites from the gut to the cochlea poses formidable technical obstacles. Additionally, the anatomical inaccessibility of the inner ear limits local tissue sampling, and peripheral fecal and blood biomarkers are insufficient to reflect authentic microenvironmental alterations within auditory tissues.

To advance the gut–ear axis from correlational observations to clinically actionable mechanisms, several research priorities deserve attention. Large-scale prospective cohort studies with serial assessments of gut microbiome composition, systemic inflammatory markers, and audiovestibular function are needed to establish temporal trajectories and to distinguish causal drivers from secondary consequences. Such cohorts should include diverse populations and adopt standardized protocols for audiometric and vestibular evaluation, with sufficient follow-up duration to capture disease onset and progression.

Mechanistic dissection at the strain and molecular level is another priority. While 16S rRNA-based surveys have provided valuable taxonomic insights, future investigations should apply metagenomic and metabolomic integration to identify functional microbial modules that actively influence inner ear homeostasis, such as butyrate synthesis pathways or GABA-producing gene clusters. Definitive causal validation will require gnotobiotic animal models colonized with defined microbial consortia, combined with targeted metabolite supplementation or genetic perturbation experiments.

Patient-stratified clinical trials are also essential to translate these insights into practice. Given the substantial heterogeneity in both gut microbiota composition and audiovestibular phenotypes, future interventional studies should stratify participants by baseline microbiome signatures, inflammatory endotypes, or disease duration rather than treating audiovestibular disorders as uniform entities. Probiotics or prebiotics may prove more effective in patients with baseline SCFA deficiency or elevated systemic inflammation, whereas individuals with distinct microbial profiles may require alternative strategies.

Technological advances in inner ear imaging and minimally invasive sampling, together with progress in barrier biology, may eventually permit direct assessment of cochlear microenvironmental responses to gut-derived signals. Such capabilities would help confirm the in vivo relevance of pathways currently extrapolated from the gut–brain axis.

In summary, these research directions should help determine whether the gut-ear axis can be translated into measurable benefits for patients with audiovestibular disorders. The current evidence base, though growing, remains insufficient for clinical application, and rigorous investigation along these lines is required before any microbiome-based strategies can be recommended in routine practice.

## 7. Conclusions

In summary, gut dysbiosis contributes to audiovestibular disorders through immune-mediated barrier disruption, metabolite imbalance, and neurotransmitter crosstalk. Current evidence is predominantly correlative and limited by cross-sectional designs, small sample sizes, and a lack of human causal data. Future research should prioritize longitudinal cohort studies, multi-omics integration, and mechanism-driven clinical trials to translate gut–ear axis correlations into targeted therapies.

## Figures and Tables

**Table 1:** Gut Microbiota-Auditory Disorder Interactions: Related flora, mechanisms, treatments and evidence.

Category	Sensorineural Hearing Loss (SNHL)	Otitis Media (OM)	Tinnitus	Ménière’s Disease (MD)
Microbiota Changes	Protective SCFA producing anaerobes like *Lachnospiraceae* and *Intestinimonas* are linked to lower SNHL risk [28]; *Bifidobacterium* is associated with higher SNHL risk [29]	Nasopharyngeal commensals (*Corynebacterium*, *Dolosigranulum*) reduced [30]; pathogens (NTHi, *M. catarrhalis*) increased [31]; in chronic OM: *H. influenzae*, *Alloiococcus*, *Pseudomonas* [31]	Reduced diversity; Weissella lower in severe tinnitus [32]; higher Actinobacteria linked to higher risk [33]	Butyrate producers (*Oscillospiraceae*, *Butyricimonas*) reduced [34]; Butyricicoccus increased with disease duration
Core Mechanisms	High-fat diet causes leaky gut and systemic inflammation that reaches the cochlea, leading to ICAM1, IL-6Rα, TLR2 upregulation and macrophage infiltration, oxidative stress, and hair cell damage [18]	Eustachian tube problems [35]; virus-bacteria synergy; overactive mucosal immunity	Gut inflammation with TNF-α, IL-1β, and IL-6 damages the blood-labyrinth barrier, causing central auditory neuroinflammation, low GABA, and microglial activation	SCFA deficiency weakens the gut barrier, resulting in low-grade inflammation and neuroinflammation in vestibular nuclei, low serotonin, and poor central compensation
Related Treatments	SPIOCA nanoparticles (oral, barrier repair) [17]; Mediterranean diet (omega-3); probiotics	*oral S. salivarius* K12 [36]; xylitol; 1,8-cineole	GABA boosting probiotics (Bifidobacterium) [37]; high fiber diet; TNF-α inhibitors (animal only) [38]	taVNS [39]; gastrodin(no gut harm) [40];
Key Evidence	SPIOCA lowered ABR threshold by 15 dB in noise-damaged mice [17]; Mendelian randomization shows specific food preferences and gut bacteria influence SNHL risk [41]; protective effects of *Lachnospiraceae* and *Intestinimonas* [41]; risk association with Bifidobacterium [29]	Nasal spray cut OM recurrence by 42% (RCT) [17]; probiotic formula cut acute OM by 50% [42]; xylitol reduces OM by 20–40% [43]	TNF-α knockout or inhibition reduces tinnitus-like behavior in animals [38]; probiotics raise cortical GABA [32]	taVNS speeds vestibular compensation (clinical trial) [39]; gastrodin effective without damaging gut [40]

Abbreviations: ICAM1, Intercellular Adhesion Molecule 1; IL-6Rα, interleukin-6 Receptor Alpha; SPIOCA, Superparamagnetic Iron Oxide Nanoparticle Assembly; NTHi, non-typeable *Haemophilus influenzae;* taVNS, Transcutaneous Auricular Vagus Nerve Stimulation; ABR, Auditory Brainstem Response.

**Table 2:** Summary of evidence linking gut microbiota to audiovestibular disorders, classified by study design and evidence level (OCEBM 2011) [44].

Disease	Key Finding	Study Design	OCEBM Level	Evidence Strength
Sensorineural Hearing Loss (SNHL)	*Lachnospiraceae UCG001* and *Intestinimonas* are associated with lower SNHL risk [28]	Mendelian randomization (MR)	3	Moderate
	*Bifidobacterium* abundance linked to higher SNHL risk [28]	MR	3	Moderate
	Fecal microbiota transplantation from enteritis mice elevates auditory threshold in recipients [19]	Animal experiment	5	Low
	High-fat diet induces cochlear inflammation [18,45]	Animal experiment	5	Low
	Omega-3/Mediterranean diet inversely associated with hearing loss [39,46,47,48,49,50]	Cross-sectional/Cohort	4	Low to Moderate
Otitis Media (OM)	Probiotics reduce AOM risk by 20% [42,51]	Meta-analysis of RCTs	1a	High
	Nasal spray with alpha-hemolytic streptococci reduces OM recurrence by 42% [17]	RCT	1b	High
	Xylitol reduces OM episodes by 20–40% [17]	Meta-analysis of RCTs	1a	High
	*Turicibacter* abundance correlated with infection frequency in rAOM [43,52]	Cross-sectional	4	Low
	Gut microbiome composition shows limited differences between rAOM and controls [52]	Cross-sectional	4	Low
Tinnitus	Reduced gut microbial diversity and increased Firmicutes/Bacteroidetes ratio in tinnitus patients [32]	Cross-sectional + Metabolomics	4	Low to Moderate
	Microbiota–metabolite model achieves AUC 0.94–0.96 for tinnitus discrimination [32]	Cross-sectional	4	Low
	Actinobacteria abundance associated with higher tinnitus risk [53]	MR	3	Moderate
	Microbiota–brain connectivity mediation via salience/default mode networks [53,54]	Cross-sectional (rfMRI)	4	Low
Ménière’s Disease (MD)	*Butyricicoccus* abundance positively correlated with disease duration [34]	Cross-sectional	4	Low
	Reduced SCFA producers in UL mouse model [55]	Animal experiment	5	Low
Benign Paroxysmal Positional Vertigo (BPPV)	Eight microbial taxa show protective association with BPPV [56]	MR	3	Moderate
	Mediation via obesity and HbA1c for specific taxa-BPPV association [56]	MR + Mediation analysis	3	Moderate
Vestibular Migraine (VM)	Probiotics: little or no effect on vertigo episodes or quality of life [57]	Cochrane systematic review (RCTs)	1a	High
	Altered gut microbiota and serum metabolome in VM-like rat model [58]	Animal experiment	5	Low

Abbreviations: OCEBM, Oxford Centre for Evidence-Based Medicine; MR, Mendelian randomization; AOM, acute otitis media; rAOM, recurrent acute otitis media; rfMRI, Resting-state functional MRI; AUC, area under the curve; UL, unilateral labyrinthectomy; HbA1c, glycated hemoglobin A1c.

## Data Availability

No new data were created or analyzed in this study. Data sharing is not applicable to this article.
